# Supplementary material for: Breeding Strategies to Optimize Effective Population Size in Low Census Captive Populations: The Case of Gazella cuvieri
Source: Animals (Basel). 2021 May 27;11(6):1559. doi: 10.3390/ani11061559 (PMC8226440; doi:10.3390/ani11061559)
Supplement: Supplementary file 1 [file animals-11-01559-s001.zip › animals-1197306-supplementary.pdf]

Table S1. Evolution of effective population size throughout 20 generations of each strategy in the population of Almería and their standard errors. a) Mixed strategies. b) Strategies that minimize the coancestry between the parents. c) Strategies that minimize the coancestry of the offspring.

Generation	**mix 1-99**	**mix 5-95**	**mix 50-50**	**mix 95-5**	**mixf 1-99**	**mixf 5-95**	**mixf 50-50**	**mixf 95-5**
**1**	14.44 ±0.001	14.87 ±0.000	14.97 ±0.000	15.01 ±0.000	15.02 ±0.001	15.37 ±0.000	15.87 ±0.000	16.44 ±0.001
**2**	14.65 ±0.001	14.74 ±0.001	14.91 ±0.001	15.09 ±0.001	14.51 ±0.001	15.12 ±0.000	15.24 ±0.000	11.42 ±0.007
**3**	14.84 ±0.001	14.91 ±0.001	15.08 ±0.002	15.24 ±0.001	14.98 ±0.001	15.50 ±0.001	15.63 ±0.001	10.95 ±0.005
**4**	15.10 ±0.001	15.12 ±0.002	15.29±0.002	15.04 ±0.002	15.27 ±0.002	15.64 ±0.001	15.59 ±0.001	11.31 ±0.004
**5**	15.27 ±0.002	15.32 ±0.002	15.46±0.002	14.80 ±0.002	15.26 ±0.002	15.88 ±0.002	15.64 ±0.002	11.66 ±0.004
**6**	15.46 ±0.002	15.56 ±0.002	15.65±0.002	14.60 ±0.002	15.41 ±0.002	15.94 ±0.002	15.68 ±0.003	11.81 ±0.004
**7**	15.61 ±0.002	15.70 ±0.002	15.69±0.003	14.42 ±0.002	15.61 ±0.002	16.12 ±0.002	15.79 ±0.003	11.50 ±0.004
**8**	15.82 ±0.002	15.87 ±0.002	15.88±0.003	14.26 ±0.002	15.76 ±0.002	16.30 ±0.002	15.88 ±0.003	11.36 ±0.004
**9**	15.91 ±0.002	15.89 ±0.002	15.97±0.003	14.25 ±0.002	15.85 ±0.003	16.39 ±0.002	15.94 ±0.003	11.40 ±0.004
**10**	15.97 ±0.002	16.04 ±0.002	16.12±0.003	14.07 ±0.002	15.88 ±0.002	16.54 ±0.002	15.97 ±0.003	11.43 ±0.004
**11**	16.12 ±0.002	16.21 ±0.002	16.20±0.003	14.12 ±0.002	16.01 ±0.003	16.66 ±0.003	16.01 ±0.003	11.41 ±0.004
**12**	16.25 ±0.002	16.32 ±0.003	16.29±0.003	14.00 ±0.002	16.13 ±0.003	16.67 ±0.003	16.09 ±0.003	11.40 ±0.004
**13**	16.33 ±0.002	16.37 ±0.003	16.36±0.003	13.93 ±0.002	16.31 ±0.003	16.77 ±0.003	16.10 ±0.003	11.38 ±0.004
**14**	16.39 ±0.002	16.51 ±0.003	16.41±0.003	13.80 ±0.002	16.30 ±0.003	16.82 ±0.003	16.10 ±0.003	11.37 ±0.004
**15**	16.47 ±0.002	16.56 ±0.003	16.48±0.003	13.83 ±0.002	16.40 ±0.003	16.99 ±0.003	16.13 ±0.004	11.29 ±0.004
**16**	16.56 ±0.003	16.63 ±0.002	16.49±0.003	13.73 ±0.002	16.49 ±0.003	17.03 ±0.003	16.15 ±0.004	11.36 ±0.004
**17**	16.63 ±0.002	16.68 ±0.003	16.51±0.003	13.71 ±0.002	16.51 ±0.003	17.06 ±0.003	16.17 ±0.004	11.40 ±0.004
**18**	16.72 ±0.003	16.73 ±0.003	16.56±0.003	13.63 ±0.002	16.63 ±0.003	17.15 ±0.003	16.24 ±0.003	11.35 ±0.004
**19**	16.70 ±0.002	16.76 ±0.002	16.61 ±0.003	13.66 ±0.002	16.70 ±0.003	17.23 ±0.003	16.29 ±0.003	11.29 ±0.004
**20**	16.80 ±0.002	16.83 ±0.002	16.65 ±0.003	13.54 ±0.002	16.77 ±0.002	17.33 ±0.003	16.33 ±0.003	11.27 ±0.004

a)

Generation	Ff	F0	F1	ΔF0	ΔF1	mFm0	mFm1	ΔmΔFΔm0	ΔmΔFΔm1
**1**	16.50 ±0.000	15.04 ±0.000	15.03 ±0.001	14.98 ±0.000	15.02 ±0.001	14.82 ±0.000	14.89 ±0.000	14.83 ±0.000	14.84 ±0.000
**2**	8.77 ± 0.002	15.12 ±0.001	15.03 ±0.001	14.96 ±0.001	15.02 ±0.001	14.52 ±0.002	14.82 ±0.001	14.33 ±0.002	14.75 ±0.001
**3**	9.49 ±0.002	15.05 ±0.001	15.02 ±0.001	14.61 ±0.001	14.80 ±0.001	13.59 ±0.003	14.64 ±0.002	13.54 ±0.003	14.46 ±0.002
**4**	10.06 ±0.002	14.65 ±0.003	14.94 ±0.002	14.25 ±0.002	14.84 ±0.002	13.02 ±0.003	14.44 ±0.002	13.07 ±0.003	14.34 ±0.002
**5**	10.21 ±0.003	14.32 ±0.003	14.82 ±0.002	13.77 ±0.003	14.69 ±0.002	12.61 ±0.003	14.36 ±0.003	12.57 ±0.003	14.10 ±0.003
**6**	9.35 ±0.004	13.97 ±0.003	14.70 ±0.003	13.58 ±0.003	14.71 ±0.002	12.17 ±0.003	14.27 ±0.003	12.21 ±0.003	13.92 ±0.003
**7**	9.09 ±0.004	13.81 ±0.003	14.53 ±0.003	13.44 ±0.003	14.65 ±0.003	11.88 ±0.003	14.13 ±0.003	11.92 ±0.003	13.95 ±0.003
**8**	9.10 ±0.004	13.55 ±0.003	14.50 ±0.003	13.30 ±0.003	14.58 ±0.003	11.59 ±0.003	14.14 ±0.003	11.57 ±0.003	13.85 ±0.003
**9**	9.01 ±0.004	13.44 ±0.003	14.52 ±0.003	13.09 ±0.003	14.52 ±0.003	11.36 ±0.003	14.04 ±0.003	11.46 ±0.003	13.69 ±0.003
**10**	8.83 ±0.004	13.34 ±0.003	14.41 ±0.003	12.98 ±0.003	14.49 ±0.003	11.14 ±0.003	14.02 ±0.003	11.26 ±0.003	13.65 ±0.003
**11**	8.76 ±0.004	13.21 ±0.003	14.38 ±0.003	12.84 ±0.003	14.46 ±0.003	10.98 ±0.004	13.95 ±0.004	11.06 ±0.003	13.65 ±0.003
**12**	8.58 ±0.003	13.07 ±0.003	14.35 ±0.003	12.76 ±0.003	14.40 ±0.002	10.79 ±0.004	13.97 ±0.004	10.94 ±0.003	13.50 ±0.004
**13**	8.51 ±0.003	13.01 ±0.003	14.29 ±0.003	12.70 ±0.003	14.43 ±0.003	10.66 ±0.004	13.98 ±0.004	10.79 ±0.003	13.49 ±0.004
**14**	8.48 ±0.003	12.84 ±0.003	14.27 ±0.003	12.55 ±0.003	14.37 ±0.003	10.55 ±0.004	13.90 ±0.004	10.67 ±0.003	13.47 ±0.004
**15**	8.44 ±0.003	12.82 ±0.003	14.22 ±0.003	12.38 ±0.003	14.38 ±0.003	10.47 ±0.004	13.83 ±0.004	10.56 ±0.003	13.40 ±0.004
**16**	8.41 ±0.003	12.66 ±0.003	14.25 ±0.003	12.37 ±0.003	14.30 ±0.003	10.34 ±0.003	13.79 ±0.003	10.48 ±0.003	13.41 ±0.003
**17**	8.39 ±0.003	12.55 ±0.003	14.18 ±0.003	12.30 ±0.003	14.26 ±0.003	10.26 ±0.003	13.80 ±0.003	10.36 ±0.003	13.45 ±0.003
**18**	8.31 ±0.003	12.55 ±0.003	14.24 ±0.003	12.26 ±0.003	14.23 ±0.003	10.22 ±0.003	13.79 ±0.004	10.27 ±0.003	13.41 ±0.003
**19**	8.28 ±0.002	12.42 ±0.003	14.23 ±0.003	12.20 ±0.003	14.22 ±0.003	10.16 ±0.003	13.71 ±0.003	10.21 ±0.003	13.38 ±0.003
**20**	8.24 ±0.002	12.36 ±0.003	14.18 ±0.003	12.13 ±0.003	14.18 ±0.003	10.11 ±0.003	13.67 ±0.003	10.16 ±0.003	13.33 ±0.003

b)

Generation	C0	C1	ΔC0	ΔC1	Cf	ΔCf
**1**	13.30 ±0.001	13.27 ±0.001	13.21 ±0.001	13.09 ±0.001	14.77 ±0.001	14.53 ±0.001
**2**	13.50 ±0.002	13.47 ±0.002	13.47 ±0.002	13.42 ±0.002	13.92 ±0.002	14.00 ±0.002
**3**	13.80 ±0.002	13.73 ±0.002	13.61 ±0.002	13.81 ±0.002	14.32 ±0.002	14.38 ±0.002
**4**	13.94 ±0.002	13.89 ±0.002	13.98 ±0.002	14.10 ±0.002	14.41 ±0.002	14.63 ±0.002
**5**	14.22 ±0.002	14.27 ±0.002	14.16 ±0.002	14.22 ±0.002	14.94 ±0.003	14.80 ±0.002
**6**	14.45 ±0.002	14.46 ±0.002	14.38 ±0.002	14.50 ±0.002	15.15 ±0.003	15.20 ±0.002
**7**	14.62 ±0.002	14.64 ±0.002	14.68 ±0.002	14.54 ±0.002	15.32 ±0.003	15.29 ±0.002
**8**	14.84 ±0.002	14.74 ±0.002	14.68 ±0.002	14.86 ±0.002	15.51 ±0.003	15.36 ±0.003
**9**	15.09 ±0.002	15.04 ±0.002	15.03 ±0.002	14.89 ±0.003	15.81 ±0.003	15.66 ±0.003
**10**	15.24 ±0.002	15.14 ±0.002	15.04 ±0.002	15.06 ±0.003	15.93 ±0.003	15.77 ±0.003
**11**	15.34 ±0.003	15.18 ±0.002	15.37±0.003	15.13 ±0.003	15.87 ±0.003	15.84 ±0.003
**12**	15.46 ±0.003	15.27 ±0.002	15.41 ±0.003	15.13 ±0.003	16.14 ±0.002	16.09 ±0.003
**13**	15.35 ±0.003	15.50 ±0.002	15.56 ±0.003	15.30 ±0.003	16.10 ±0.003	16.15 ±0.003
**14**	15.51 ±0.003	15.60 ±0.002	15.60 ±0.003	15.39 ±0.003	16.19 ±0.003	16.28 ±0.003
**15**	15.57 ±0.003	15.63 ±0.002	15.59 ±0.003	15.49 ±0.003	16.39 ±0.003	16.25 ±0.003
**16**	15.73 ±0.003	15.67 ±0.002	15.74 ±0.003	15.60 ±0.003	16.37 ±0.004	16.35 ±0.003
**17**	15.74 ±0.003	15.88 ±0.002	15.88 ±0.003	15.70 ±0.003	16.48 ±0.004	16.33 ±0.003
**18**	15.88 ±0.002	15.84 ±0.002	15.95 ±0.002	15.62 ±0.003	16.39 ±0.004	16.47 ±0.003
**19**	15.89 ±0.003	16.06 ±0.003	16.05 ±0.003	15.77 ±0.002	16.44 ±0.004	16.48 ±0.003
**20**	15.98 ±0.003	16.10 ±0.003	16.02 ±0.003	15.82 ±0.003	16.64 ±0.004	16.50 ±0.003

c)

Table S2. Evolution of effective population size throughout 20 generations of each strategy in the population of La Lajita and their standard errors. a) Mixed strategies. b) Strategies that minimize the coancestry between the parents. c) Strategies that minimize the coancestry of the offspring.

Generation	**mix 1-99**	**mix 5-95**	**mix 50-50**	**mix 95-5**	**mixf 1-99**	**mixf 5-95**	**mixf 50-50**	**mixf 95-5**
**1**	9.01 ±0.001	9.02 ±0.001	9.15±0.001	9.26±0.001	9.14 ±0.001	9.32 ±0.001	9.51±0.000	9.79±0.000
**2**	9.80 ±0.001	9.79 ±0.001	9.88±0.001	9.93±0.001	9.87±0.001	10.03 ±0.001	10.11 ±0.001	8.06±0.006
**3**	10.25 ±0.001	10.38 ±0.001	10.41±0.001	10.02±0.002	10.30±0.001	10.42±0.001	10.53±0.001	8.01±0.002
**4**	10.60 ±0.001	10.72 ±0.001	10.69±0.001	10.12±0.002	10.65±0.002	10.81±0.001	10.77±0.002	8.50±0.002
**5**	11.01 ±0.001	11.10±0.001	11.06±0.001	10.28±0.002	11.03±0.002	11.23±0.002	11.08±0.002	9.01±0.002
**6**	11.34 ±0.001	11.46 ±0.001	11.36±0.002	10.49±0.002	11.40±0.002	11.54±0.002	11.38±0.002	9.21±0.002
**7**	11.65 ±0.001	11.80±0.002	11.68±0.002	10.66±0.002	11.74±0.002	11.86±0.002	11.67±0.002	9.14±0.002
**8**	11.96 ±0.001	12.12 ±0.002	11.97±0.002	10.81±0.002	12.06±0.002	12.14±0.002	11.94 ±0.003	9.20±0.003
**9**	12.23 ±0.002	12.37 ±0.002	12.21±0.003	10.96±0.002	12.34±0.002	12.50±0.002	12.14±0.003	9.37±0.003
**10**	12.49 ±0.002	12.66±0.002	12.49±0.002	11.12±0.002	12.63±0.002	12.68±0.002	12.34±0.003	9.48±0.004
**11**	12.76 ±0.002	12.92±0.002	12.66±0.002	11.15±0.002	12.81±0.002	12.89±0.002	12.52±0.003	9.52±0.003
**12**	12.96 ±0.002	13.14±0.002	12.89±0.002	11.24±0.002	12.93±0.003	13.15 ±0.002	12.75±0.003	9.56±0.003
**13**	13.16 ±0.002	13.34±0.002	13.10±0.003	11.37±0.002	13.03±0.002	13.34±0.002	12.92±0.003	9.66±0.003
**14**	13.36 ±0.002	13.53±0.002	13.28±0.002	11.46±0.002	13.30±0.002	13.55±0.00	13.11±0.003	9.68±0.003
**15**	13.57 ±0.002	13.71±0.002	13.46±0.002	11.50±0.002	13.50±0.003	13.69±0.003	13.28±0.003	9.76±0.003
**16**	13.73 ±0.002	13.89±0.002	13.64±0.002	11.62±0.002	13.65±0.002	13.87±0.003	13.41±0.003	9.80 ±0.003
**17**	13.88 ±0.002	14.04±0.002	13.80±0.002	11.68±0.002	13.82±0.002	14.00±0.003	13.52±0.003	9.80 ±0.003
**18**	14.04 ±0.002	14.21±0.002	13.94±0.002	11.77±0.002	13.92±0.003	14.10±0.003	13.62±0.003	9.86±0.003
**19**	14.23 ±0.002	14.35±0.002	14.07±0.002	11.82±0.002	14.07±0.003	14.24±0.003	13.76±0.003	9.96±0.003
**20**	14.39 ±0.002	14.47±0.002	14.19±0.002	11.82±0.002	14.21±0.002	14.43±0.002	13.89±0.002	10.00±0.002

a)

Generation	**Ff**	**F0**	**F1**	**ΔF0**	**ΔF1**	**mFm0**	**mFm1**	**ΔmΔFΔm0**	**ΔmΔFΔm1**
**1**	9.78±0.000	9.21±0.000	8.96±0.001	9.26±0.000	8.95 ±0.001	9.12±0.001	8.90±0.001	9.25±0.001	8.95±0.000
**2**	7.06±0.001	9.92±0.001	9.60±0.001	9.87±0.001	9.64 ±0.001	9.83±0.001	9.61±0.001	9.76±0.001	9.61±0.001
**3**	7.76±0.000	9.86±0.002	10.10±0.001	9.68±0.002	10.11±0.001	9.89±0.001	10.12±0.001	9.65±0.001	10.04±0.002
**4**	8.36±0.002	9.98±0.002	10.44±0.001	9.84±0.002	10.45±0.001	9.80±0.002	10.32±0.002	9.61±0.002	10.39±0.002
**5**	8.36±0.003	10.09±0.002	10.77±0.002	9.88±0.002	10.72±0.002	9.75±0.002	10.69±0.002	9.57±0.002	10.72±0.002
**6**	7.95±0.003	10.15±0.002	11.05±0.001	10.02±0.002	10.99±0.002	9.74±0.002	10.97±0.002	9.58±0.002	10.94±0.002
**7**	7.98±0.003	10.23±0.002	11.24±0.002	10.11±0.002	11.22±0.002	9.68±0.002	11.20±0.002	9.61±0.002	11.16±0.002
**8**	7.97±0.003	10.26±0.003	11.50±0.002	10.17±0.002	11.47±0.002	9.64±0.002	11.34±0.002	9.58±0.002	11.39±0.002
**9**	7.89±0.003	10.29±0.003	11.69±0.002	10.25±0.002	11.67±0.002	9.60±0.002	11.55±0.002	9.53±0.002	11.56±0.002
**10**	7.85±0.003	10.39±0.003	11.85±0.002	10.28±0.002	11.82±0.002	9.60±0.002	11.73±0.002	9.49±0.002	11.75±0.002
**11**	7.82±0.003	10.45±0.003	12.06±0.002	10.30±0.002	12.02±0.002	9.61±0.002	11.90±0.003	9.48±0.002	11.90±0.002
**12**	7.79±0.003	10.49±0.003	12.16±0.002	10.35±0.002	12.20±0.002	9.56±0.002	12.08±0.003	9.47±0.002	12.03±0.002
**13**	7.84±0.003	10.52±0.003	12.37±0.002	10.41±0.002	12.34±0.002	9.53±0.003	12.25±0.003	9.42±0.002	12.17±0.002
**14**	7.79±0.003	10.54±0.002	12.53±0.002	10.41±0.002	12.53±0.002	9.50±0.002	12.36±0.002	9.44±0.002	12.22±0.002
**15**	7.74±0.002	10.55±0.002	12.66±0.002	10.46±0.002	12.64±0.002	9.49±0.002	12.50±0.003	9.41±0.002	12.32±0.002
**16**	7.76±0.003	10.58±0.002	12.78±0.002	10.48±0.002	12.78±0.002	9.47±0.002	12.55±0.003	9.43±0.002	12.43±0.002
**17**	7.68±0.002	10.61±0.002	12.92±0.002	10.50±0.002	12.89±0.002	9.46±0.002	12.62±0.003	9.40±0.002	12.57±0.002
**18**	7.64±0.002	10.64±0.002	13.05±0.002	10.51±0.002	12.96±0.002	9.43±0.002	12.76±0.003	9.38 ±0.002	12.70±0.002
**19**	7.63±0.002	10.68±0.002	13.14±0.002	10.53±0.002	13.09±0.002	9.42±0.002	12.86±0.003	9.35±0.002	12.78±0.002
**20**	7.63±0.002	10.71±0.002	13.23±0.002	10.57±0.002	13.20±0.002	9.40±0.002	12.95±0.003	9.30±0.002	12.89±0.002

b)

Generation	C0	C1	ΔC0	ΔC1	Cf	ΔCf
**1**	8.59±0.001	8.47±0.001	8.61±0.001	8.50±0.001	8.94±0.001	8.89±0.001
**2**	9.27±0.002	8.99±0.002	9.18±0.001	8.96±0.002	9.51±0.002	9.36±0.002
**3**	9.68±0.002	9.48±0.002	9.61±0.002	9.45±0.002	9.68±0.002	9.86±0.002
**4**	10.09±0.002	9.77±0.002	10.07±0.002	9.85±0.002	10.20±0.002	10.30±0.002
**5**	10.41±0.002	10.18±0.002	10.29±0.002	10.18±0.002	10.39±0.003	10.65±0.002
**6**	10.81±0.002	10.67±0.003	10.67±0.002	10.58±0.002	10.84±0.003	10.98±0.002
**7**	11.09±0.002	10.83±0.002	10.98±0.002	10.82±0.003	11.15±0.003	11.29±0.002
**8**	11.34±0.002	11.04±0.002	11.32±0.002	11.23±0.002	11.51±0.003	11.58±0.002
**9**	11.70±0.002	11.38±0.003	11.62±0.002	11.41±0.003	11.62±0.003	12.01±0.002
**10**	11.90±0.002	11.67±0.002	11.87±0.002	11.66±0.002	12.04±0.002	12.14±0.002
**11**	12.10±0.002	11.99±0.002	12.08±0.002	11.85±0.003	12.21±0.003	12.40±0.002
**12**	12.45±0.002	12.19±0.002	12.29±0.002	12.11±0.002	12.50±0.003	12.58±0.002
**13**	12.60±0.002	12.36±0.003	12.54±0.002	12.35±0.002	12.60±0.003	12.86±0.002
**14**	12.79±0.002	12.59±0.002	12.67±0.002	12.61±0.003	12.78±0.003	13.14±0.002
**15**	12.95±0.002	12.69±0.003	12.82±0.002	12.68±0.002	12.98±0.003	13.21±0.002
**16**	13.20±0.002	12.91±0.002	13.01±0.002	12.91±0.002	13.18±0.003	13.44±0.002
**17**	13.26±0.002	13.02±0.003	13.21±0.002	13.00±0.002	13.35±0.003	13.58±0.002
**18**	13.51±0.002	13.11±0.003	13.38±0.002	13.09±0.003	13.49±0.003	13.68±0.002
**19**	13.67±0.002	13.35±0.003	13.58±0.002	13.28±0.002	13.64±0.003	13.75±0.002
**20**	13.74±0.002	13.42±0.003	13.64±0.002	13.44±0.002	13.78±0.003	13.99±0.002

c)
